# Association of Food Insecurity With Allostatic Load Among Older Adults in the US

**DOI:** 10.1001/jamanetworkopen.2021.37503

**Published:** 2021-12-07

**Authors:** Tae-Young Pak, GwanSeon Kim

**Affiliations:** 1 Department of Consumer Science and Convergence Program for Social Innovation, Sungkyunkwan University, Seoul, South Korea; 2 College of Agriculture, Arkansas State University, Jonesboro

## Abstract

**Importance:**

Allostatic overload, a biomarker of wear and tear, could be the potential pathway through which food insecurity leads to increased morbidity risk.

**Objective:**

To assess the association of food insecurity with allostatic load (AL) among US adults aged 50 years or older.

**Design, Setting, and Participants:**

A multiwave longitudinal cohort study was conducted using data from the 2006 to 2014 waves of the Health and Retirement Study in a national cohort study setting. The data comprise 26 509 person-years observations from 14 394 noninstitutionalized individuals aged 50 years or older during the study period. Data were analyzed from September 1 to December 14, 2020.

**Exposures:**

Moderate food insecurity (not enough money to buy the food needed) and severe food insecurity (reduced food intake due to financial constraints) measured at the household level.

**Main Outcomes and Measures:**

The AL score (0-9, with higher scores indicating a greater risk of physiologic dysregulation) and binary indicators of dysregulated inflammatory (C-reactive protein), cardiovascular (systolic and diastolic blood pressure, pulse rate, and cystatin C), and metabolic (hemoglobin A_1c_, body mass index, waist-to-height ratio, total cholesterol to high-density lipoprotein cholesterol ratio) systems.

**Results:**

Of 14 394 participants included in the analysis, the median age was 60 (IQR, 56-69) years, 8143 (56.6%) were women, 517 (3.6%) were moderately food insecure, and 804 (5.6%) were severely food insecure. In adjusted models, the incidence rate of AL was 1.05 (95% CI, 1.00-1.09) times higher for the participants with moderate food insecurity and 1.11 (95% CI, 1.07-1.15) times higher for the participants with severe food insecurity, compared with those who were food secure. The increased incidence rate of AL among participants with severe food insecurity was associated with C-reactive protein level (odds ratio [OR], 1.22; 95% CI, 1.04-1.44), cystatin C level (OR, 1.23; 95% CI, 1.01-1.51), hemoglobin A_1c_ level (OR, 1.27; 95% CI, 1.01-1.59), body mass index (OR, 1.84; 95% CI, 1.41-2.40), waist-to-height ratio (OR, 1.54; 95% CI, 1.26-1.88), and total to high-density lipoprotein cholesterol ratio (OR, 1.32; 95% CI, 1.10-1.59) inflated to the high-risk range. The interaction between moderate food insecurity and Supplemental Nutrition Assistance Program (SNAP) enrollment (β = −0.18; *P* = .001) and the interaction between severe food insecurity and SNAP enrollment (β = −0.09; *P* = .02) were associated with a reduction in AL.

**Conclusions and Relevance:**

In this national cohort study of US adults aged 50 years or older, food insecurity was associated with higher AL, mainly through dysregulation of the inflammatory and metabolic systems. SNAP enrollment may modify this association between food insecurity and AL.

## Key Points

**Question:**

Is food insecurity associated with allostatic load among older US adults?

**Findings:**

In this cohort study of 14 394 older adults (≥50 years) taken from the Health and Retirement Study, food insecurity was associated with a higher level of allostatic load through dysregulated inflammatory and metabolic systems. In addition, Supplemental Nutrition Assistance Program enrollment was shown to attenuate the association of allostatic load with moderate food insecurity.

**Meaning:**

The findings of this study suggest that food insecurity is associated with increased allostatic load, and a food assistance program could mitigate this association.

## Teaser

This cohort study evaluates allostatic load in individuals with no, moderate, or severe food insecurity.

## Introduction

Food insecurity, defined as limited access to nutritionally adequate and safe foods owing to financial constraints,^1^ poses a substantial challenge to the maintenance of good health. In 2018, 7.3 million older individuals in the US were food insecure, representing 10% of adults aged 60 years or older in the US.^2^ About one-third of individuals with food insecurity exhibited multiple indications of disrupted eating patterns and reduced food intake,^2^ which is associated with worse health and higher rates of health care use.^3,4,5,6^ The prevalence of food insecurity has been more pronounced for persons in racial and ethnic minority groups, older individuals with disabilities, low-income households, and those infected with COVID-19^2,7,8,9^—the population subgroups that carry relatively higher health risks.

An emerging body of literature has documented associations between food insecurity and chronic disease in individuals at older ages, including diabetes, heart disease, hypertension, kidney disease, obesity, and pulmonary disease.^3,4,6,10,11,12,13,14,15^ Studies have reported that food-insecure older adults in the US and Canada were subject to higher mortality risk and shorter life spans than their food-secure peers.^16,17,18,19,20^ Despite considerable evidence of its health consequences, little is known about the mechanisms by which food insecurity causes adverse health outcomes.^21^ It is thought that food insecurity makes people replace healthy diets with inexpensive, high-calorie diets, potentiating visceral fat accumulation and obesity-related chronic conditions.^22,23^ Furthermore, repeated episodes of hunger were shown to incite susceptibility to infection^24^ or increase the risk of peripheral insulin resistance—a precursor to diabetes—among persons with food insecurity.^5,25^

Chronic stress associated with food insecurity offers an alternative physiologic pathway leading to chronic disease: the allostatic load (AL) pathway.^26^ Allostatic load refers to the physiologic wear and tear that the body experiences due to chronic stress exposure and repeated activation of the adaptive stress response.^27^ Chronic activation in terms of duration or frequency can result in dysregulations across multiple physiologic systems and ultimately makes the body lose its ability to maintain homeostasis.^28,29,30^ The physiologic consequences of AL have been used to explain how socioeconomic disadvantages “get under the skin” in the form of chronic illness.^31,32,33^ In terms of food insecurity, a study of Puerto Rican adults aged 45 to 75 years found an association between food insecurity and AL mediated by dysregulations in the neuroendocrine and inflammatory systems.^26^

The objective of this study was to examine the association between food insecurity and AL using data from a national cohort study of US adults aged 50 years or older. Using biomarker data from the Health and Retirement Study (HRS), we assessed longitudinal associations between food insecurity and AL, as measured by the inflammatory (C-reactive protein), cardiovascular (systolic and diastolic blood pressure, pulse rate, and cystatin C), and metabolic (hemoglobin A_1c_ [HbA_1c_,] body mass index, waist-to-height ratio, total cholesterol to high-density lipoprotein cholesterol ratio) systems.^32,34,35,36,37^ In addition, we tested for the hypothesis that participation in the Supplemental Nutrition Assistance Program (SNAP) may help moderate the association between food insecurity and AL. This study builds on related studies^26^ by using a longitudinal study design and providing generalizable findings on how food insecurity may be associated with an increased risk of chronic disease.

## Methods

### Data Source and Study Sample

The HRS is a nationally representative, prospective cohort study of US residents aged 50 years or older, conducted by the University of Michigan’s Institute for Social Research.^38^ The survey has been fielded every 2 years since 1992 to collect data on participants’ health, demographic, and socioeconomic characteristics. Written informed consent was obtained from all participants prior to each interview. This study is a secondary analysis of the deidentified, public-release version of the HRS; therefore, the institutional review board at the Sungkyunkwan University exempted this research from further review and approval. This study followed the Strengthening the Reporting of Observational Studies in Epidemiology (STROBE) reporting guideline for cohort studies.

From 2006 to 2014, the HRS collected anthropometric measurements and blood-based biomarkers through an enhanced face-to-face interview. The enhanced face-to-face interview was administered to half of a randomly selected sample in one wave and to the other half in the next wave; thus, consenting participants submitted biomarkers every other wave on a rotating schedule. During the enhanced face-to-face interview, trained interviewers conducted tests of physical performance and collected saliva and dried blood spots using specialized tools.^39^ The dried blood spot samples were assayed for 5 biomarkers: C-reactive protein, cystatin C, hemoglobin A_1c_, total cholesterol, and high-density lipoprotein cholesterol. Details on recruitment and data collection have been published.^40^

The study sample was restricted to individuals in the 2006-2014 HRS, with information on biomarkers, food insecurity, SNAP, and other control variables (eAppendix in the Supplement). After listwise deletion of missing data, our analytic sample comprised 26 509 person-years observations from 14 394 baseline participants aged 50 to 100 years (eFigure in the Supplement).

### Food Insecurity and SNAP Participation

Food insecurity was assessed with 2 items adapted from a 2-item screener for household food insecurity.^41^ Participants were first asked “In the last 2 years, have you always had enough money to buy the food you need?”; those who answered no to this question were further asked, “In the last 12 months, did you ever eat less than you felt you should because there wasn't enough money to buy food?” The responses to these screener questions lead to an ordinal measure of food insecurity, which assigns 0 to participants who answered yes to the first question (food security), 1 to participants who gave a no response to both questions (moderate food insecurity), and 2 to participants who answered no to the first question and yes to the second question (severe food insecurity).

SNAP enrollment was determined by 2 questions, “Did you (or other family members who were living here) receive government food stamps at any time since the previous interview / in the last two years?” and “Are you (or other family members who are living [here/there]) still receiving food stamps?.” A response of yes to both questions is assumed as confirmation of SNAP enrollment.^42,43^ All questions on food insecurity and SNAP enrollment were addressed by one spouse in each household, designated as a representative spouse who answers household-level questions.

### Allostatic Load

Allostatic load is based on 9 biomarkers indicating dysregulation of the inflammatory system (C-reactive protein), cardiovascular system (systolic and diastolic blood pressure, pulse, and cystatin C), and metabolic system (hemoglobin A_1c_, body mass index, waist-to-height ratio, and total cholesterol to high-density lipoprotein cholesterol ratio). For each biomarker, we constructed a binary indicator of high risk based on the cutoff values commonly accepted in the clinical literature (Table 1).^32,34,35,36^ The composite AL score was constructed by summing the number of binary indicators above the defined cutoff. Thus, the AL score ranges from 0 to 9, with a higher score indicating a greater risk of physiologic dysregulation. Our operationalization follows the procedures of the MacArthur Studies of Successful Aging report,^37^ which selected biological parameters on theoretical grounds and clinical validity.

**Table 1.** Baseline Characteristics of Biomarkers and Cutoff Points for High-Risk Values in 14 394 Older Adults

System and biomarker	Mean (SD)	>Cutoff, %	High-risk cutoff points
Inflammatory			
C-reactive protein, μg/mL	3.9 (7.1)	34.2	>3
Cardiovascular			
Systolic BP, mm Hg	134.2 (21.2)	26.5	Mean of 3 measurements >140
Diastolic BP, mm Hg	82.4 (11.8)	15.8	Mean of 3 measurements >90
Pulse rate, bpm	70.6 (11.4)	19.0	Mean of 3 measurements >80
Cystatin C, mg/L	1.1 (0.5)	16.7	≥1.29
Metabolic			
HbA_1c_, %	5.8 (1.0)	13.3	≥6.5 or taking medicine
BMI	28.5 (5.8)	12.4	≥35 (morbid obesity)
Waist-to-height ratio	0.6 (0.1)	49.0	>0.6
Cholesterol ratio	4.0 (1.2)	43.3	Total to HDL cholesterol ratio ≥5.92 or using prescription medications to lower cholesterol level

Abbreviations: BMI, body mass index (calculated as weight in kilograms divided by height in meters squared); BP, blood pressure; HbA_1c_, hemoglobin A_1c_; HDL, high-density lipoprotein.

SI conversion factors: To convert C-reactive protein to milligrams per liter, multiply by 10; HbA_1c_ to proportion of total hemoglobin, 0.01.

### Covariates

Age, sex, race and ethnicity (Hispanic, non-Hispanic African American, non-Hispanic White, and Other), educational level background (less than college or college graduate), marital status (separated, divorced, widowed, never married, or married), number of living children, smoking (currently smoking or not smoking), binge drinking (≥4 alcoholic drinks per occasion, <4 alcoholic drinks per occasion, or no drinking), number of private health insurance coverages, out-of-pocket (OOP) medical spending, employment status (working or not working), household income (sum of earnings, pensions and annuities, government transfers, and capital income earned by spouses), household wealth (the sum of cash and cash-equivalent assets, stocks, bonds, certificate of deposits, retirement savings, vehicles, real estate, and private businesses, minus unsecured debt), cohort fixed effects, and year-of-survey dummies were included as covariates in the analyses. Race and ethnicity were reported by survey participants and included in regressions to account for differences in AL by race and ethnicity.

Additionally considered covariates included total daily calories and total daily intake of calcium, iron, magnesium, potassium, sodium, copper, and manganese. Further adjusting our main regressions to the nutrition variables would help uncover whether the main results were due to malnutrition or other possible mediators associated with food insecurity have been considered. The calorie and nutrient data were obtained from the 2013 Health Care and Nutrition Study^44^ and match-merged with the 2014 HRS wave.

### Statistical Analysis

Three-level mixed models with random intercepts specified for the household and census division levels were estimated. Specifically, the AL score for an individual *i* in household *j*, nested in census division *k,* was related to the individual- and household-level covariates, including both measures of food insecurity and SNAP enrollment. To address our research questions, the following regression models were specified: (1) a mixed-effects Poisson regression of the AL score on food insecurity and covariates, (2) a mixed-effects logistic regression of the binary indicator of the high-risk biomarker on food insecurity, and (3) a mixed-effects Poisson regression of the AL score on food insecurity, SNAP, food insecurity × SNAP, and covariates. Model 1 corresponds to our main research issue (association between food insecurity and AL), model 2 serves to identify individual biomarkers responsible for the main results, and model 3 examines whether SNAP enrollment moderates the association between food insecurity and AL.

Across all regressions, adjusted incidence rate ratios and adjusted odds ratios were reported along with their 95% CIs. Coefficients with *P* < .05 for a 2-sided test were considered statistically significant. The *mepoisson* and *melogit* commands in Stata, version 16 MP (StataCorp LLC) were used to estimate the regression models. Data cleaning and analyses were performed from September 1 to December 14, 2020.

## Results

### Study Sample

Table 2 reports the baseline characteristics of 14 394 HRS participants included in this study. The study sample consisted of 8143 women (56.6%) and 6251 men (43.4%), with 3202 college graduates (22.2%). The race and ethnicity groups comprised 2524 African American (17.5%), 1815 Hispanic, (12.6%), and 9578 White (66.5%) individuals; the Other category included 477 (3.3%) American Indian, Alaska Native, Asian, Hawaiian Native, or Pacific Islander individuals. The median age was 60 (IQR, 56-69) years, and 1032 individuals (7.2%) were SNAP beneficiaries. By food security status, 13 073 participants (90.8%) were food secure, 517 (3.6%) were moderately food insecure, and 804 (5.6%) were severely food insecure. A total of 9112 (63.3%) were fully retired or not working, and 5282 (36.7%) were employed. The mean (SD) of the AL score for the full sample was 2.27 (1.68), which was higher for the moderately food insecure participants (2.53 [1.70]) and the severely food insecure participants (2.74 [1.81]), compared with the food-secure participants (2.23 [1.67]). The follow-up data are summarized in eTable 1 in the Supplement.

**Table 2.** Baseline Distribution of Demographic Characteristics and Allostatic Load by Food Security Among 14 394 Health and Retirement Study Participants

Characteristics^a^	Participants, No. (%)				AL score, mean (SD)^b^
	Food insecure		Food secure (n = 13 073)	All (n = 14 394)	
	Moderate (n = 517)	Severe (n = 804)			
Person-years	881	1345	24 283	26 509	NA
AL, mean (SD)	2.53 (1.70)	2.74 (1.81)	2.23 (1.67)	2.27 (1.68)	NA
SNAP					
Not received	410 (79.3)	538 (66.9)	12 414 (95.0)	13 362 (92.8)	2.23 (1.66)
Received	107 (20.7)	266 (33.1)	659 (5.0)	1032 (7.2)	2.98 (1.85)
Age, median (IQR), y	59 (55-64)	56 (54-61)	61 (56-70)	60 (56-69)	NA
Sex					
Female	315 (60.9)	519 (64.6)	7309 (55.9)	8143 (56.6)	2.24 (1.70)
Male	202 (39.1)	285 (35.4)	5764 (44.1)	6251 (43.4)	2.30 (1.66)
Race and ethnicity					
Hispanic	102 (19.7)	157 (19.5)	1556 (11.9)	1815 (12.6)	2.55 (1.69)
Non-Hispanic African American	173 (33.5)	302 (37.6)	2049 (15.7)	2524 (17.5)	2.95 (1.79)
Non-Hispanic White	228 (44.1)	312 (38.8)	9038 (69.1)	9578 (66.5)	2.15 (1.64)
Other^c^	14 (2.7)	33 (4.1)	430 (3.3)	477 (3.3)	2.28 (1.65)
Educational level					
Less than college	444 (85.9)	738 (91.8)	10 010 (76.6)	11 192 (77.8)	2.44 (1.70)
College graduate	73 (14.1)	66 (8.2)	3063 (23.4)	3202 (22.2)	1.81 (1.53)
Marital status					
Never married	31 (6.0)	98 (12.2)	516 (3.9)	645 (4.5)	2.37 (1.78)
Separated, divorced, widowed	182 (35.2)	357 (44.4)	3540 (27.1)	4079 (28.3)	2.48 (1.70)
Married	304 (58.8)	349 (43.4)	9017 (69.0)	9670 (67.2)	2.18 (1.66)
No. of living children, mean (SD)	3.23 (2.24)	3.04 (2.13)	2.88 (1.86)	2.90 (1.89)	NA
Binge drinking					
None	494 (95.6)	724 (90.0)	12 436 (95.1)	13 654 (94.9)	2.27 (1.69)
Binge	23 (4.4)	80 (10.0)	637 (4.9)	740 (5.1)	2.30 (1.58)
Smoking					
Not currently	409 (79.1)	490 (60.9)	11 207 (85.7)	12 106 (84.1)	2.26 (1.68)
Currently	108 (20.9)	314 (39.1)	1866 (14.3)	2288 (15.9)	2.34 (1.69)
No. of health insurance plans, mean (SD)	0.51 (0.53)	0.29 (0.54)	0.76 (0.59)	0.73 (0.60)	NA
Employment status					
Not working	335 (64.8)	533 (66.3)	8244 (63.1)	9112 (63.3)	2.53 (1.69)
Working	182 (35.2)	271 (33.7)	4829 (36.9)	5282 (36.7)	1.94 (1.62)
OOP medical spending, mean (SD), $^d^	3.0 (5.2)	3.0 (6.1)	2.8 (4.8)	2.8 (4.9)	NA
Household income, mean (SD), $^d^^,^^e^	36.6 (103.8)	15.9 (26.9)	41.1 (67.7)	39.7 (68.1)	NA
Household wealth, mean (SD), $^d^^,^^f^	218.6 (830.3)	13.0 (161.1)	344.5 (1035.4)	323.6 (1006.1)	NA

Abbreviations: AL, allostatic load; NA, not applicable; OOP, out-of-pocket; SNAP, Supplemental Nutrition Assistance Program.

^a^ Statistics are weighted using the individual and household weights provided by the RAND Health and Retirement Study.

^b^ Scores range from 0 to 9, with higher scores indicating a greater risk of physiologic dysregulation.

^c^ Other races included Alaska Native, American Indian, Asian, Hawaiian Native, and Pacific Islander.

^d^ Dollars in thousands.

^e^ Household income comprises the sum of earnings, pensions and annuities, government transfers, and capital income earned by spouses.

^f^ Household wealth comprises the sum of cash and cash-equivalent assets, stocks, bonds, certificate of deposits, retirement savings, vehicles, real estate, and private businesses, minus unsecured debt.

### Food Insecurity and AL

The mixed-effects multivariable Poisson regressions of AL on food insecurity and covariates are presented in Table 3. The baseline regression showed a 15% (incidence rate ratio [IRR], 1.15; 95% CI, 1.10-1.20) increase in the incidence rate of AL for being in moderate food insecurity and a 27% (IRR, 1.27; 95% CI, 1.22-1.31) increase for being in severe food insecurity. The IRR was estimated to be 1.07 (95% CI, 1.03-1.12) for moderate food insecurity and 1.16 (95% CI, 1.12-1.20) for severe food insecurity when the model accounted for demographic factors, cohort dummies, and year-of-survey fixed effects. The fully adjusted model showed that the incidence rate of AL was 1.05 (95% CI, 1.00-1.09) times higher for the moderately food-insecure group and 1.11 (95% CI, 1.07-1.15) times higher for the severely food-insecure group compared with the food-secure respondents. When analyses were restricted to each sex, severe food insecurity was associated with AL (IRR, 1.10; 95% CI, 1.05-1.16 for women; IRR, 1.07; 95% CI, 1.01-1.15 for men), but moderate food insecurity was no longer associated with AL (IRR, 1.04; 95% CI, 0.98-1.10 for women; IRR, 1.06; 95% CI, 0.99-1.14 for men). Severe food insecurity was associated with a higher level of AL when the full model included additional controls of total calories and total nutrient intakes (IRR, 1.13; 95% CI, 1.01-1.26) (eTable 2 in the Supplement).

**Table 3.** Association of Moderate and Severe Food Insecurity With Allostatic Load, Mixed-Effects Poisson Regression Results

Variable	Full sample		Women		Men	
	IRR (95% CI)	*P* value	IRR (95% CI)	*P* value	IRR (95% CI)	*P* value
**Baseline model^a^**						
Food insecurity						
Moderate	1.15 (1.10-1.20)	<.001	1.18 (1.11-1.25)	<.001	1.13 (1.05-1.21)	.001
Severe	1.27 (1.22-1.31)	<.001	1.32 (1.26-1.38)	<.001	1.17 (1.10-1.25)	<.001
**Demographic factors–adjusted model^b^**						
Food insecurity						
Moderate	1.07 (1.03-1.12)	.002	1.06 (1.00-1.12)	.04	1.08 (1.01-1.17)	.03
Severe	1.16 (1.12-1.20)	<.001	1.16 (1.11-1.21)	<.001	1.11 (1.04-1.18)	.002
**SES-adjusted model^c^**						
Food insecurity						
Moderate	1.09 (1.05-1.14)	<.001	1.10 (1.04-1.16)	.001	1.09 (1.01-1.17)	.03
Severe	1.15 (1.11-1.19)	<.001	1.17 (1.12-1.22)	<.001	1.10 (1.03-1.17)	.005
**Fully adjusted model^d^**						
Food insecurity						
Moderate	1.05 (1.00-1.09)	.03	1.04 (0.98-1.10)	.21	1.06 (0.99-1.14)	.09
Severe	1.11 (1.07-1.15)	<.001	1.10 (1.05-1.16)	<.001	1.07 (1.01-1.15)	.03

Abbreviations: AL, allostatic load; IRR, incidence rate ratio; SES, socioeconomic status.

^a^ Adjusted for cohort dummies and year-of-survey fixed effects.

^b^ Adjusted for age, sex, race and ethnicity, educational background, marital status, number of living children, smoking, binge drinking, cohort dummies, and year-of-survey fixed effects.

^c^ Adjusted for number of health insurance plans, out-of-pocket medical spending, employment status, household income, household wealth, cohort dummies, and year-of-survey fixed effects.

^d^ Adjusted for age, sex, race and ethnicity, educational background, marital status, number of living children, smoking, binge drinking, number of health insurance plans, out-of-pocket medical spending, employment status, household income, household wealth, cohort dummies, and year-of-survey fixed effects.

### Food Insecurity and Indicators of High-Risk Biomarkers

Table 4 presents the results of the mixed-effects multivariable logistic regression for high-risk biomarkers. We found that severe food insecurity was associated with high-risk levels of C-reactive protein (odds ratio [OR], 1.22; 95% CI, 1.04-1.44), cystatin C (OR, 1.23; 95% CI, 1.01-1.51), hemoglobin A_1c_ (OR, 1.27; 95% CI, 1.01-1.59), body mass index (OR, 1.84; 95% CI, 1.41-2.40), waist-to-height ratio (OR, 1.54; 95% CI, 1.26-1.88), and cholesterol ratio (OR, 1.32; 95% CI, 1.10-1.59), controlling for a full set of covariates (demographic factors, socioeconomic status, cohort, and time-fixed effects). Moderate food insecurity was associated with C-reactive protein level (OR, 1.24; 95% CI, 1.03-1.50) and waist-to-height ratio (OR, 1.29; 95% CI, 1.03-1.62), but not with other high-risk biomarkers. The high-risk biomarkers of the cardiovascular system were generally not associated with food insecurity (except cystatin C level).

**Table 4.** Association of Moderate and Severe Food Insecurity With High-Risk Biomarkers, Mixed-Effects Logistic Regression Results

System	Dependent variable^b^	Independent variable, food insecurity^a^			
		Moderate		Severe	
		OR (95% CI)	*P* value	OR (95% CI)	*P* value
Inflammatory	C-reactive protein	1.24 (1.03-1.50)	.03	1.22 (1.04-1.44)	.01
Cardiovascular	Systolic BP	1.02 (0.86-1.24)	.75	1.10 (0.94-1.28)	.26
	Diastolic BP	1.03 (0.83-1.27)	.80	1.08 (0.91-1.29)	.36
	Pulse	1.03 (0.84-1.27)	.77	1.07 (0.90-1.27)	.44
	Cystatin C	1.00 (0.79-1.27)	.97	1.23 (1.01-1.51)	.04
Metabolic	HbA_1c_	1.09 (0.84-1.41)	.52	1.27 (1.01-1.59)	.04
	BMI	1.20 (0.86-1.67)	.27	1.84 (1.41-2.40)	<.001
	Waist-to-height ratio	1.29 (1.03-1.62)	.03	1.54 (1.26-1.88)	<.001
	Cholesterol ratio	0.96 (0.78-1.18)	.67	1.32 (1.10-1.59)	.003

Abbreviations: BMI, body mass index; BP, blood pressure; OR, odds ratio.

^a^ Adjusted for age, sex, race and ethnicity, educational background, marital status, number of living children, smoking, binge drinking, number of health insurance plans, out-of-pocket medical spending, employment status, household income, household wealth, cohort dummies, and year-of-survey fixed effects.

^b^ Binary indicators of the biomarker being in the high-risk range.

### Moderating Effect of SNAP Enrollment

The interaction terms, moderate food insecurity × SNAP and severe food insecurity × SNAP, were added to the fully adjusted model (Table 5). The results showed the coefficient estimate of −0.18 (95% CI, −0.29 to −0.07) for moderate food insecurity × SNAP, indicating that the association between moderate food insecurity and AL was significantly attenuated conditional on SNAP enrollment. The interaction between severe food insecurity and SNAP had a statistically significant estimate of −0.09 (95% CI, −0.17 to −0.01), giving further support for the moderating effect of SNAP across varying degrees of food insecurity.

**Table 5.** Association of Moderate and Severe Food Insecurity With Allostatic Load Conditional on SNAP Enrollment, Mixed Effects Poisson Regression Results

Independent variable^b^	AL score^a^	
	β (95% CI)	*P* value
Moderate food insecurity	0.07 (0.03 to 0.12)	.003
Severe food insecurity	0.11 (0.07 to 0.16)	<.001
SNAP	0.13 (0.09 to 0.16)	<.001
Moderate food insecurity × SNAP	−0.18 (−0.29 to −0.07)	.001
Severe food insecurity × SNAP	−0.09 (−0.17 to −0.01)	.02

Abbreviations: AL, allostatic load; SNAP, Supplemental Nutrition Assistance Program.

^a^ Scores range from 0 to 9, with higher scores indicating a greater risk of physiologic dysregulation.

^b^ Adjusted for age, sex, race and ethnicity, educational background, marital status, number of living children, smoking, binge drinking, number of health insurance plans, out-of-pocket medical spending, employment status, household income, household wealth, cohort dummies, and year-of-survey fixed effects.

## Discussion

The association between food insecurity and increased risk of mortality has been robustly reported in the literature, reported in numerous studies documenting negative health outcomes among food-insecure individuals.^16,17,18,19,20^ As discussed in the Introduction, physiologic dysregulation resulting from limited food access could be an underlying mechanism through which food insecurity is biologically embedded in the tissues and organs of the body, ultimately leading to a deterioration in health. We developed a multisystemic index of AL representing the major regulatory systems and examined its association with food insecurity.

The results showed a 5% increase in AL associated with being moderately food insecure and an 11% increase associated with being severely food insecure, adjusted for demographic and socioeconomic confounders. Further analyses highlighted significant inflations of the inflammatory and metabolic biomarkers to the high-risk ranges. In addition, there was suggestive evidence that SNAP participation may mitigate the association of AL with moderate and severe food insecurity. Overall, these findings suggest that the link between food insecurity and an increased risk of chronic disease^3,4,6,11,12,13,14,15^ could be mediated by physiologic dysregulation^26^ and that appropriate intervention could help modify this association.^26,42,45,46^

A higher level of C-reactive protein, a validated biomarker of inflammation, in food-insecure individuals corroborates the evidence that food insecurity is associated with inflammation and related immune responses. Shifts in dietary patterns and the stress of being food insecure have been shown to predispose individuals with food insecurity to an inflammatory state and reduce immune function.^24,25,47^ Although acute immune response could be beneficial, repeated activation of the immune system over an extended period can cause a breakdown of immune tolerance and increase susceptibility to infections and metabolic syndromes.^48,49^ The clinical consequences of chronic inflammation include hypertension, dyslipidemia, type 2 diabetes, cardiovascular disease, chronic kidney disease, and depression,^50,51,52,53^ which are prevalent in food-insecure older adults.

Our findings are largely congruent with prior research, except for the results that food insecurity was not associated with high-risk biomarkers of the cardiovascular system. This difference could be attributed to the older study population, in which cardiovascular disease is more common regardless of food insecurity, or our longitudinal study design that captures immediate changes in cardiovascular biomarkers that arise within 1 or 2 years after an individual becomes food insecure. This finding is partly in line with Seligman et al,^5^ who reported that evidence for an association between food insecurity and hyperlipidemia was weak compared with its association with diabetes. They suggested that the association between food insecurity and hypertension could be mediated by medication adherence, which could not be considered in our study.

Examining multiple physiologic parameters offers a useful opportunity to evaluate the short-term and direct health effects of food insecurity interventions. Because physiologic parameters are able to capture an immediate health response to the intervention, they can be used as a direct performance indicator of how intervention or policy change affects beneficiaries' health. Physiologic parameters may also help identify areas in which the intervention is or is not achieving its goal, by showing physiologic parameters that are responsive to intervention. This finding can provide early evidence on whether the intervention has created the desired outcome and which aspect needs further refinement.

### Strengths and Limitations

This study has several strengths, including a nationally representative sample, collection of biomarker data through standardized procedures by trained interviewers,^40^ controlling for a large number of possible confounders, and a comprehensive assessment of food insecurity and AL in conjunction with SNAP. Use of biomarker data is also supported by the World Health Organization recommendation to shift from a disease-oriented definition of health to more objective indicators of physical capacity and functioning in research concerning older adults.^54^

The study also has limitations. First, our measure of AL does not comprise a complete set of biomarkers commonly used to assess biological dysregulation.^32^ It also assumed equal weight to each biomarker by taking the sum of binary indicators for elevated risk. A more expansive measure that taps into all components of AL and an alternative approach of aggregation needs to be established to reveal the full health consequences of food insecurity.^33^ Second, owing to data limitations, the participants have less than 2 measurements on average. Thus, our estimates might be subject to measurement error and indicate underestimated association between food insecurity and AL. Third, because this study was based on observational data, we could not establish the causality of our estimates. Future research exploiting exogenous variation in food insecurity or a randomized clinical trial may contribute to the literature by confirming causality. Fourth, our analysis was limited to the relatively short-term outcome of food insecurity. Fifth, this study could not explicitly test whether AL was a mediator between food insecurity and morbidity. Future research is warranted to examine how food-insecurity–induced AL leads to chronic conditions using data with longer follow-up periods.

## Conclusions

In this prospective cohort study based on the HRS data set, an association between AL and food insecurity was found for 14 394 US adults aged 50 to 100 years. The results showed evidence of the increased AL in food insecurity, associated with the inflammatory and metabolic biomarkers inflated to the high-risk range among participants with severe food insecurity. There was a significant interaction between SNAP enrollment and food insecurity, suggesting that SNAP may protect against the adverse health outcomes noted with food insecurity increasing AL.

## Notes

Supplement. **eAppendix.** Health and Retirement Study Datasets Used for This Study **eFigure.** Selection of the Study Sample from the Health and Retirement Study, 2006-2014 **eTable 1.** Follow-up Distribution of Demographic Characteristics and Allostatic Load by Food Security (n=12 115) **eTable 2.** Mixed Effects Poisson Regression of Food Insecurity on Allostatic Load, Adjusted for Total Calories and Nutrient Intakes (n=2689)
