# Supplementary material for: Association of Food Insecurity With Allostatic Load Among Older Adults in the US
Source: JAMA Netw Open. 2021 Dec 7;4(12):e2137503. doi: 10.1001/jamanetworkopen.2021.37503 (PMC8652609; doi:10.1001/jamanetworkopen.2021.37503)
Supplement: Supplement. — eAppendix. Health and Retirement Study Datasets Used for This Study eFigure. Selection of the Study Sample from the Health and Retirement Study, 2006-2014 eTable 1. Follow-up Distribution of Demographic Characteristics and Allostatic Load by Food Security (n=12 115) eTable 2. Mixed Effects Poisson Regression of Food Insecurity on Allostatic Load, Adjusted for Total Calories and Nutrient Intakes (n=2689) [file jamanetwopen-e2137503-s001.pdf]

Supplementary Online Content

Pak TY, Kim G. Association of food insecurity with allostatic load among older adults in the US. *JAMA Netw Open*. 2021;4(12):e2137503. doi:10.1001/jamanetworkopen.2021.37503

eAppendix. Health and Retirement Study Datasets Used for This Study

eFigure. Selection of the Study Sample from the Health and Retirement Study, 2006-2014

eTable 1. Follow-Up Distribution of Demographic Characteristics and Allostatic Load by Food Security (n=12 115)

eTable 2. Mixed Effects Poisson Regression of Food Insecurity on Allostatic Load, Adjusted for Total Calories and Nutrient Intakes (n=2689)

This supplementary material has been provided by the authors to give readers additional information about their work.

© 2021 Pak TY et al. *JAMA Network Open.*

eAppendix. Health and Retirement Study Datasets Used for This Study

1. RAND HRS longitudinal file 2016 (V1)
2. HRS blood-based biomarkers datasets from 2006 to 2014
3. HRS core dataset module I from 2006 to 2014 (for physical measures)
4. HRS core dataset module N from 2006 to 2014 (for medication intake)
5. HRS core dataset module Q from 2006 to 2014 (for food insecurity and SNAP enrollment)
6. HRS leave-behind module from 2006 to 2012 (for total daily calories and nutrient intake)

© 2021 Pak TY et al. *JAMA Network Open.*

eFigure. Selection of the Study Sample from the Health and Retirement Study, 2006-2014

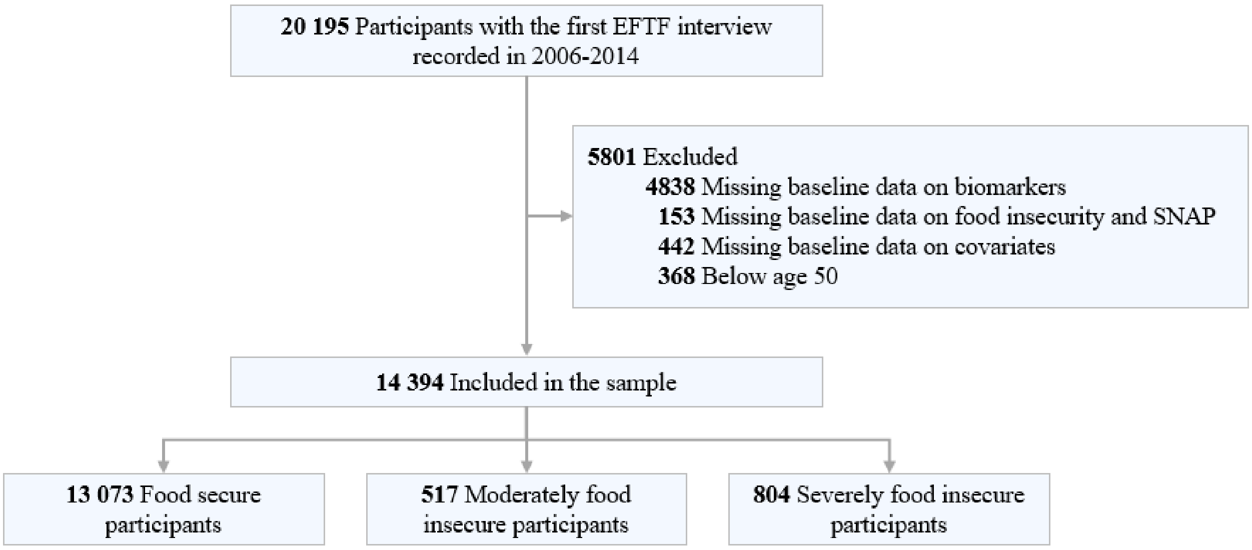

© 2021 Pak TY et al. *JAMA Network Open.*

eTable 1. Follow-Up Distribution of Demographic Characteristics and Allostatic Load by Food Security (n=12 115)

	Participants, No. (%)				
	Food insecure				
Characteristics[a]	Moderate (n=364)	Severe (n=541)	Food secure (n=11 210)	All (n=12 115)	AL score, mean (SD)[b]
AL, mean (SD)	2.75 (1.94)	3.00 (1.69)	2.36 (1.68)	2.39 (1.69)	NA
SNAP					
Not received	283 (77.7)	352 (65.1)	10 566 (94.3)	11 201 (92.5)	2.35 (1.67)
Received	81 (22.3)	189 (34.9)	644 (5.7)	914 (7.5)	3.11 (1.75)
Age, median (IQR), y	64 (12)	62 (10)	67 (14)	66 (14)	NA
Sex					
Female	237 (65.1)	375 (69.3)	6561 (58.5)	7173 (59.2)	2.34 (1.70)
Male	127 (34.9)	166 (30.7)	4649 (41.5)	4942 (40.8)	2.47 (1.67)
Race and ethnicity					
Hispanic	56 (15.4)	96 (17.7)	1035 (9.2)	1187 (9.8)	2.76 (1.69)
NH African American	120 (33.0)	181 (33.5)	1361 (12.1)	1662 (13.7)	3.05 (1.69)
NH White	175 (48.1)	243 (44.9)	8535 (76.1)	8953 (73.9)	2.30 (1.67)
Other[c]	13 (3.6)	21 (3.9)	279 (2.5)	313 (2.6)	2.58 (1.73)
Educational level					
Less than college	316 (86.8)	500 (92.4)	8342 (74.4)	9158 (75.6)	2.61 (1.68)
College graduate	48 (13.2)	41 (7.6)	2868 (25.6)	2957 (24.4)	1.89 (1.59)
Marital status					
Never married	17 (4.7)	49 (9.1)	359 (3.2)	425 (3.5)	2.64 (1.79)
Separated, divorced, widowed	178 (48.9)	278 (51.4)	3445 (30.7)	3901 (32.2)	2.61 (1.69)
Married	169 (46.4)	214 (39.6)	7406 (66.1)	7789 (64.3)	2.28 (1.67)
No. of living children, mean (SD)	2.96 (1.91)	3.22 (2.12)	2.92 (1.86)	2.93 (1.87)	NA
Binge drinking					
None	349 (95.9)	513 (94.8)	10 778 (96.1)	11 640 (96.1)	2.39 (1.69)
Binge	15 (4.1)	28 (5.2)	432 (3.9)	475 (3.9)	2.56 (1.72)
Smoking					
Not currently	300 (82.4)	359 (66.4)	10 052 (89.7)	10 711 (88.4)	2.37 (1.69)
Currently	64 (17.6)	182 (33.6)	1158 (10.3)	1404 (11.6)	2.59 (1.70)
No. of health insurance plans, mean (SD)	0.50 (0.68)	0.28 (0.48)	0.70 (0.64)	0.68 (0.64)	NA
Employment status					
Not working	277 (76.1)	425 (78.6)	8519 (76.0)	9221 (76.1)	2.57 (1.67)
Working	87 (23.9)	116 (21.4)	2691 (24.0)	2894 (23.9)	1.99 (1.66)
OOP medical spending, mean (SD), $[d]	2.8 (4.1)	4.5 (15.8)	3.0 (4.9)	3.0 (5.7)	NA
Household income, mean (SD), $[d]	28.3 (47.3)	14.2 (12.2)	43.8 (71.1)	42.2 (69.5)	NA
Household wealth, mean (SD), $[d]	148.4 (436.0)	28.1 (136.5)	410.5 (949.9)	388.5 (925.1)	NA

Abbreviation: AL, Allostatic Load; SNAP, Supplemental Nutrition Assistance Program; IQR, interquartile range; NH, non-Hispanic; OOP, out-of-pocket.

[a] Statistics are weighted using the individual and household weights provided by the RAND Health and Retirement Study.

[b] Score ranges from 0 to 9, with higher scores indicating a greater risk of physiologic dysregulation.

[c] Other races included American Indian, Alaska Native, Asian, Hawaiian Native, and Pacific Islander.

[d] Dollars in thousands.

© 2021 Pak TY et al. *JAMA Network Open.*

eTable 2. Mixed Effects Poisson Regression of Food Insecurity on Allostatic Load, Adjusted for Total Calories and Nutrient Intakes (n=2689)

		AL score[a]	
		IRR (95% CI)	*P* value
Fully adjusted[b]	Moderate food insecurity	1.06 (0.92, 1.23)	0.41
	Severe food insecurity	1.14 (1.01, 1.27)	0.03
Fully adjusted + total calories[c]	Moderate food insecurity	1.06 (0.92, 1.23)	0.42
	Severe food insecurity	1.14 (1.01, 1.27)	0.03
Fully adjusted + nutrition[d]	Moderate food insecurity	1.07 (0.93, 1.23)	0.37
	Severe food insecurity	1.13 (1.01, 1.26)	0.04

Abbreviation: AL, allostatic load; IRR, incidence rate ratio.

[a] Scores range from 0 to 9, with higher scores indicating a greater risk of physiologic dysregulation.

[b] Adjusted for age, gender, race, education background, marital status, number of living children, smoking, binge drinking, number of health insurance plans, OOP medical spending, labor supply, household income, household wealth, and cohort dummies.

[c] Adjusted for total daily calories, age, gender, race, education background, marital status, number of living children, smoking, binge drinking, number of health insurance plans, OOP medical spending, labor supply, household income, household wealth, and cohort dummies.

[d] Adjusted for total daily intake of calcium, iron, magnesium, potassium, sodium, copper, manganese, age, gender, race, education background, marital status, number of living children, smoking, binge drinking, number of health insurance plans, OOP medical spending, labor supply, household income, household wealth, and cohort dummies.

© 2021 Pak TY et al. *JAMA Network Open.*
